# The Role of Gonadotropin-Releasing Hormone (GnRH) in Endometrial Cancer

**DOI:** 10.3390/cells10020292

**Published:** 2021-02-01

**Authors:** Günter Emons, Carsten Gründker

**Affiliations:** Department of Gynecology and Obstetrics, University Medicine Göttingen, 37075 Göttingen, Germany; grundker@med.uni-goettingen.de

**Keywords:** endometrial cancer, gonadotropin releasing hormone, luteinizing hormone releasing hormone, GnRH, LHRH

## Abstract

Endometrial cancer (EC) is one of the most common gynecological malignancies. Gonadotropin releasing hormone (GnRH) is a decapeptide first described to be secreted by the hypothalamus to regulate pituitary gonadotropin secretion. In this systematic review, we analyze and summarize the data indicating that most EC express GnRH and its receptor (GnRH-R) as part of an autocrine system regulating proliferation, the cell cycle, and apoptosis. We analyze the available data on the expression and function of GnRH-II, its putative receptor, and its signal transduction. GnRH-I and GnRH-II agonists, and antagonists as well as cytotoxic GnRH-I analogs, have been shown to inhibit proliferation and to induce apoptosis in human EC cell lines in pre-clinical models. Treatment with conventional doses of GnRH-agonists that suppress pituitary gonadotropin secretion and ovarian estrogen production has become part of fertility preserving therapy of early EC or its pre-cancer (atypical endometrial hyperplasia). Conventional doses of GnRH-agonists had marginal activity in advanced or recurrent EC. Higher doses or more potent analogs including GnRH-II antagonists have not yet been used clinically. The cytotoxic GnRH-analog Zoptarelin Doxorubicin has shown encouraging activity in a phase II trial in patients with advanced or recurrent EC, which expressed GnRH-R. In a phase III trial in patients with EC of unknown GnRH-R expression, the cytotoxic GnRH doxorubicin conjugate was not superior to free doxorubicin. Further well-designed clinical trials exploiting the GnRH-system in EC might be useful.

## 1. Introduction

Endometrial cancer (EC), derived from the epithelial lining of the uterine cavity, is one of the most common female cancers. Worldwide, 382,069 new cases were diagnosed in 2018 and 89,929 women died of this disease [1]. In Western Europe and North America, EC is the fourth most common malignancy in women and the most common malignant tumor of the female genital organs [2].

The prognosis of EC is rather favorable, as about 75% of cases are diagnosed in an early stage and can be cured by surgery and in more advanced cases by additional radiotherapy and/or chemotherapy [2] resulting in a general five-year cancer-specific survival of around 80% for all stages and histological types [1,2]. The majority of EC (85%) known as the so-called type 1 cancers develop due to prolonged exposure to endogenous or exogenous estrogens in the absence of sufficient progestogen activity [3,4,5]. These type 1 EC are hormone-dependent and can be treated in early stages without surgery by endocrine manipulation, including estrogen withdrawal and/or high dose progestogens in premenopausal women who have not yet completed their families. Later in their development, these hormone-dependent EC dedifferentiate, lose expression of estrogen receptors and/or progesterone receptors, and are no longer amenable to estrogen withdrawal, anti-estrogens, or progestogens [2]. Type 2 EC do not express estrogen receptors or progesterone receptors, are not dependent on these steroids, and have a poor prognosis. They are responsible for the majority of EC-related deaths [2].

Gonadotropin releasing hormone (GnRH, also called luteinizing hormone releasing hormone, LHRH) is a hypothalamic decapeptide, regulating secretion of gonadotropins by the pituitary. By the end of the 1980s, super-active analogs of GnRH and respective depot preparations became widely available and were used for medical hypophysectomy (suppression of secretion of luteinizing hormone, LH, and follicle-stimulating hormone, FSH) leading to suppression of gonadal function in both sexes. This strategy of reversible medical castration was successfully introduced into the treatment of a variety of sex hormone-dependent diseases including prostate cancer and premenopausal breast cancer as well as endometriosis and uterine fibroids [6]. Estrogen withdrawal due to reversible medical castration through GnRH-agonists was also established as a conservative treatment of early EC and its pre-cancers in young women who wished to preserve their fertility [6].

In a variety of malignant tumors including breast, prostate, ovarian, and endometrial cancers, the expressions of GnRH and its receptors (GnRH-R) were discovered. These GnRH-R mediated direct anti-proliferative effects of agonistic and antagonistic analogs of GnRH in vitro and in nude mouse models xeno-transplanted with human cancers. Since GnRH-R are also expressed in type 2 EC, it was speculated that the treatment with GnRH-analogs might be an efficacious endocrine therapy with low toxicity for patients with EC that does not express estrogen receptors or progesterone receptors [6]. Respective clinical trials showed some activity of this approach. Finally, GnRH-analogs coupled with cytotoxic molecules were developed for targeted therapies through the GnRH-R on the surface of cancer cells. This approach has provided encouraging results in patients with EC (see below).

This systematic review summarizes the present knowledge on the GnRH-system in EC and its clinical application. In addition, we make suggestions for future research, which are both basic and clinical on this topic.

## 2. Methods

We performed a systematic literature search on the Pub Med database using the search term ((endometrial cancer) OR (endometrial carcinoma)) AND ((gonadotropin releasing hormone) OR (luteinizing hormone releasing hormone)) with an unrestricted time frame, yielding 345 results (search date 10 July 2020). The abstracts and full papers were screened independently by both authors, excluding 271 publications since they were not related to the topic, were double publications, or not in English. The remaining 74 manuscripts on original studies are described and discussed in this review. Due to the heterogeneity and the limitations of the clinical data, a meta-analysis was not appropriate.

## 3. Results

### 3.1. Expression of Receptors for GnRH in EC

Specific high affinity receptors for GnRH in EC were first described by Skralovic et al. in 1990. Using [^125^I, D-Trp^6^] GnRH as a radio-ligand, they found specific high affinity binding in 24 of 31 (77%) EC. In six of eight poorly differentiated or undifferentiated EC, high affinity GnRH binding was detected. The mathematical analysis of the binding data was consistent with a single class of high affinity, non-cooperative receptors (Kd 9.88 ± 4.59 nM, Bmax 0.70 ± 0.14 pM/mg membrane protein). No differences of incidence and binding capacity (0.01–3.8 pM/mg membrane protein) were found between well differentiated, moderately well differentiated, and poorly differentiated EC [7].

A year later, our group described a specific low affinity/high capacity binding site for GnRH in 12 of 12 EC [8]. In 1993, we detected an additional specific high affinity, low capacity binding site for [^125^I, D-Trp^6^] GnRH in human EC cell lines HEC-1A (derived from a moderately differentiated EC, estrogen receptor negative), and Ishikawa (derived from an EC expressing estrogen and progesterone receptors). Kds were 5.7 nM (HEC-1A) and 4.2 nM (Ishikawa). The respective GnRH binding capacities were 78 fmol/10^6^ cells and 29 fmol/10^6^ cells, respectively [9]. Petersen et al., using radiolabeled native GnRH or [D-Lys^6^]-GnRH, failed to detect GnRH-R in the HEC-1A cell line [10]. Using [^3^H] GnRH as a ligand, Imai et al. detected GnRH-R in RL 95-2 and HHUA EC cancer lines (Kd 2.38 ± 0.86 nM) and in 16 of 18 well differentiated and 4 of 7 poorly differentiated EC specimens (Kd 5.89 ± 3.59 nM). Bmax = 1.8 ± 0.95 pmol/mg membrane protein [11]. In addition, they found the mRNA for the GnRH-R in both cell lines and all EC samples where specific binding sites were detected.

Chatzaki et al. found low levels of GnRH-R mRNA transcripts but no high affinity GnRH binding in Ishikawa and HEC-1A cells and low affinity binding in three of eight EC tissues [12]. Shibata and colleagues showed the expression of mRNA for GnRH-R in HHUA human EC cell line [13]. Borri et al. failed to detect the expression of mRNA for the GnRH-R in HEC-1A and HEC-1B cell lines by Northern blot analysis and RT-PCR [14]. Ohta et al. detected mRNA for GnRH-R in human EC cell lines Ishikawa, EIIL, HEC1A, HEC6, HEC50, and HEC59 [15]. Kim et al. demonstrated the expression of mRNA for GnRH-R in human EC cell line CUME-1 [16]. Noci et al. using RT-PCR detected GnRH-R in 5/7 EC specimens [17].

We sequenced the complementary DNA of the GnRH-R from position 31 to position 1204, covering the complete coding region (position 56 to position 1041) in Ishikawa and HEC-1A EC cell lines. There were neither mutations nor splice variants of the GnRH-R transcript [18]. We detected high affinity GnRH binding sites (Kd 1.5 to 8.2 nM) and mRNA for the GnRH-R in EC cell lines HEC-1B, KLE, and AN-3-CA. In the endometrial cancer cell line, MFE-296 GnRH-R mRNA was found but no specific binding of GnRH detected [19]. In five EC biopsy samples including type 2 cancers, we detected specific high-affinity/low-capacity GnRH binding sites as well as the mRNA for the GnRH-R. In another Type 2 EC, neither GnRH-R nor their mRNA were found [19]. Using RT-PCR, Nagai et al. detected GnRH-R expression in the HEC-1A cell line and in 36 of 38 (95%) EC samples as well as in two samples of atypical endometrial hyperplasia (AEH), which is a precancer for EC [20]. Yang et al. found high affinity/low capacity binding sites in Ishikawa cells [21]. Engel et al. detected GnRH-R in HEC-1A and RL-95-2 EC cell lines by RT-PCR, Western blot analysis, and radio-ligand binding assays [22]. Using a monoclonal GnRH-R antibody, Jeon et al. analyzed paraffin-embedded tissue blocks from 141 endometrial cancer patients with a 10% cut off level for GnRH-R positivity. Seventy specimens (49.6%) stained as GnRH-R positive. Tumor grade, histio-type, and disease stage were not associated with GnRH-R positivity [23].

Jankowska et al. detected GnRH-R in 9 of 12 well differentiated EC using RT-PCR and immunohistochemistry [24]. In the context of a multicenter phase 2 clinical trial, we analyzed tumor samples from 48 patients with recurrent or metastatic EC for the expression of GnRH-R using immunohistochemistry. Forty-four (92.4%) of these EC were GnRH-R positive (range of positive cells: 30–90%, median: 70%, mean: 69 ± 21%) [25]. Ten of the 44 GnRH-R positive tumors were type 2 EC (serous or clear cell) [25]. The expression of GnRH-R in EC cell lines Ishikawa and ECC as well as in three EC tissue samples was demonstrated by Wu et al. using immunohistochemistry and knockdown with small interfering RNA [26,27]. Hao et al. found 16 of 20 cases of EC samples (80%) positive for GnRH-R by immunohistochemistry [28].

### 3.2. Expression of GnRH in EC

Using a specific antibody, we detected GnRH-immunoactivity in extracts of Ishikawa (426 ± 84 fmol/10^6^ cells) and HEC-1A (368 ± 41 fmol/10^6^ cells) EC cell lines [29]. GnRH bioactivity of these extracts was assessed in rat pituitary cells in the culture. The release of luteinizing hormone (LH) induced by these samples corresponded dose-dependently to that obtained with comparable amounts of authentic GnRH. The expression of mRNA for GnRH by Ishikawa and HEC-1A EC cells was demonstrated by RT-PCR, a restriction enzyme-, and Southern blot analysis [29]. Chatzaki et al. detected mRNA for GnRH in Ishikawa and HEC-1A cell lines, but not secretion of immunoreactive GnRH. In 1 of 10 primary cultures of EC tissues, GnRH mRNA and immunoreactivity were found [12]. Furui et al. detected GnRH (0.13 ± 0.074 pg/mg protein) in 12 EC samples using a radio-immunoassay and a high-performance liquid chromatography. Nine of these EC samples expressed GnRH-R [30]. Jankowska et al. demonstrated the presence of GnRH in 12 of 12 EC samples by means of RT-PCR and immunohistochemistry [24].

### 3.3. Effects of GnRH, GnRH-Agonists, and GnRH-Antagonists on the Proliferation of EC Cells

The proliferation of Ishikawa and HEC-1A EC cell lines was time and dose dependently inhibited by the GnRH agonist [D-Trp^6^] GnRH (Triptorelin) and the GnRH-antagonist Cetrorelix [9]. The anti-proliferative effects of both analogs were significant in the Ishikawa cell line at 10 pM (86%) and 1 nM in the HEC-1A cell line (80%) and maximal at 10 µM: Ishikawa 52% and HEC-1A 62% of the respective controls [9].

Kleinman et al. found that the GnRH-antagonist Cetrorelix inhibited basal and insulin like growth factor-I (IGF-I) induced growth of Ishikawa cells [31]. Peterson et al. observed no inhibition of growth of HEC-1A cells hetero-transplanted into nude mice by treatment with GnRH agonist leuprorelin [10]. Chatzaki et al. observed no effects of GnRH agonists triptorelin and goserelin on in vitro proliferation of Ishikawa and HEC-1A cells [12]. We found that GnRH agonist triptorelin dose-dependently reduced basal and epidermal growth factor (EGF) induced proliferation of HEC-1A EC cancer cell line under serum-free and phenol red-free conditions [32]. Sica and colleagues found the GnRH agonist triptorelin and leuprorelin to be ineffective on basal growth of the Ishikawa cell line but to counteract and even suppress estrogen-induced proliferation [33]. Shibata et al. reduced the proliferation of the HHUA human EC cell line by treatment with the GnRH agonist buserelin to 60% of control [13]. Borri et al. described significant anti-proliferative effects of GnRH agonist leuprorelin and of GnRH antagonist antide in HEC-1A and HEC-1B EC cell lines. [14]. Ohta et al. demonstrated an inhibition of proliferation by Triptorelin in human EC cell lines Ishikawa, EIIL, HEC1A, 6, 50, and 59 [15]. Noci and colleagues treated primary cultures from nine human EC for 96 h with 1 µM of GnRH agonist leuprorelin or GnRH antagonist cetrorelix and observed median growth inhibitions of 14.3% (leuprorelin, range 46.4–2.4%) and 27.5% (cetrorelix, 60.9–22.7%) [17]. Nagai et al. found a significant inhibition of proliferation of HEC-1A cells after treatment with 1 µM leuprorelin [20].

Zhao and colleagues observed a dose-dependent inhibition of proliferation of the HEC-1A cell line by triptorelin [34]. Park et al. described a dose-dependent inhibition of proliferation of HEC-1A EC cells with 10 and 100 nM native GnRH [35]. In ovarian cancer cell lines EFO-21 and EFO-27 that had been shown to express high affinity GnRH-R and to secrete GnRH, treatment with an antiserum to GnRH in neutralizing concentrations significantly increased proliferation by 21%, while treatment with native GnRH even at very low concentrations (1 pM) had no effect or an anti-proliferative effect. At higher concentrations of GnRH (≥10 pM), significant inhibition of proliferation was observed [36].

### 3.4. Signal Transduction and Intracellular Actions of GnRH in EC

Kleinman et al. demonstrated inhibitory effects of GnRH antagonist cetrorelix on basal and Insulin like growth factor (IGF)-induced growth of Ishikawa EC cells. This GnRH analog also completely inhibited IGF-II release from these cells [31]. Estradiol (E_2_) partially abolished the inhibitory effects of cetrorelix on the proliferation of Ishikawa cells. The growth of HEC-1A cells was not influenced by the antagonist. The GnRH agonist buserelin had no significant effect on both cell lines. The growth inhibitory effects of cetrorelix on the Ishikawa cell line was associated with an induction of apoptosis [37].

Our group showed that, in HEC-1A and Ishikawa EC cell lines, the GnRH-agonist triptorelin had no effects on GnRH-signaling mechanisms known from the pituitary gonadotrophs, i.e., phospholipase C, protein kinase C, and adenylate cyclase [38]. The proliferation of HEC-1A cells in serum/phenol red free medium was significantly increased by epidermal growth factor (EGF). This mitogenic effect of EGF was dose-dependently reduced by triptorelin. Expression of EGF-receptors was not influenced. However, net tyrosine phosphorylation induced by EGF (1 nM) was nearly completely abrogated by the addition of 10 µM triptorelin or pre-incubation with 100 nM of the GnRH agonist for 48 h. This inhibitory effect of triptorelin on EGF-induced net tyrosine phosphorylation was partly antagonized by sodium vanadate, which is an inhibitor of phosphotyrosine phosphatase. The EGF-induced activation of mitogen-activated protein kinase/extracellular signal related kinase (MAP-kinase/ERK) was completely suppressed in HEC-1A cells by pretreatment with triptorelin [32]. We could show that, in Ishikawa and HEC-1A cell lines, EGF-induced c-fos expression was dose-dependently reduced to base line levels by 100 nM of GnRH agonist triptorelin or antagonist cetrorelix [38]. Both the G protein αi and G protein αq were expressed in Ishikawa and HEC-1A cell lines and coupled to GnRH-R. Inhibition of EGF-induced c-fos expression through GnRH was, however, mediated through pertussis toxin (PTX) sensitive G protein αi. GnRH agonist triptorelin antagonized PTX-catalyzed APD-ribosylation of G protein αi. In HEC-1A and Ishikawa cells, triptorelin stimulated phospho-tyrosine phosphatase activity, which is an effect blocked by PTX. EGF induced autophosphorylation of EGF receptors was reduced by 45–63% after GnRH-agonist (100 nM) treatment.

**Figure 1:**
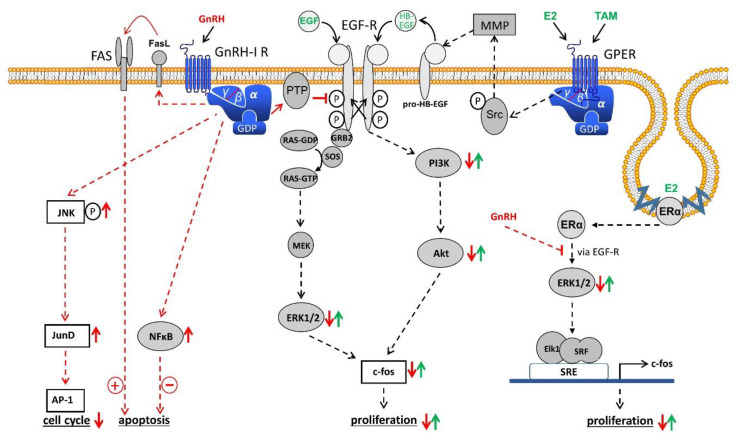
Gonadotropin releasing hormone (GnRH) receptor signal transduction in endometrial cancer (EC). Binding of GnRH-I and GnRH-I agonists causes G-protein αi-mediated activation of phospho-tyrosine phosphatase (PTP), resulting in dephosphorylated EGF-receptor (EGF-R) and inhibition of EGF-R signal transduction through the ERK1/2 or the PI3K/AKT pathway leading to a reduction of proliferation. GnRH-induced activation of PTP also inhibits the signaling cascade of G-protein coupled estrogen receptor (GPER) and estrogen receptor α (ERα) through transactivation of EGF-R. In addition, GnRH induces activation of NFκB to reduce and FAS-ligand to increase apoptosis. Finally, GnRH agonists activate the JNK/AP-1 pathway, resulting in an increased G0/1 phase of the cell cycle and decreased DNA-synthesis. Akt, protein kinase B (PKB). AP-1, activator protein-1. E2, estradiol. EGF, epidermal growth factor. ERK1/2, p44/42 mitogen-activated protein (MAP) kinase. FAS, Fas receptor. FasL, Fas ligand. GDP, guanosindiphosphate. GRB2, growth factor receptor-bound protein 2. GTP, guanosintriphosphate. HB-EGF, heparin-binding EGF-like growth factor. JNK, c-Jun N-terminale kinase. JunD, transcription factor JunD. MEK, mitogen-activated protein kinase kinase (MAP2K). MMP, matrix *metalloproteinase*. NFkB, nucleus factor kB. PI3K, phosphoinositide 3-kinase. RAS, G-protein rat sarcoma. SOS, guanine nucleotide exchange factor ”son of sevenless“. SRE, serum response element. Src, tyrosine kinase cellular sarcoma. SRF, serum response factor. TAM, tamoxifen.

This inhibitory effect of GnRH was abrogated using the phosphotyrosine phosphatase (PTP) inhibitor vanadate [18] (Figure 1). In addition, GnRH-analogs activated nucleus factor kappa-B, protecting the cancer cells from apoptosis and activator protein-1 (AP-1) through PTX-sensitive G protein αi and expression of c-Jun and activation of c-Jun phosphorylation [39] (Figure 1). The proliferation of estrogen receptor α (ERα) and estrogen receptor β (ERβ) positive EC cell lines (KLE and HEC-1B) but not of ERα negative/ERβ positive cell lines (Ishikawa, HEC-1A) was significantly stimulated by treatment with E_2_ (10 nM). This mitogenic effect of E_2_ was dose-dependently inhibited by triptorelin. E_2_ induced activation of the serum response element (SRE) and expression of the immediate early response gene c-fos was blocked by the GnRH agonist [40] (Figure 1).

In our hands, native GnRH, GnRH-agonist triptorelin, and GnRH antagonist cetrorelix reduced apoptosis in the human EC cell line Ishikawa, induced by the cytotoxic agent doxorubicin and by UV light through activation of nucleus factor-κB (NFκB) [41]. Doxorubicin induced apoptosis in HEC-1A and Ishikawa human EC cell lines were markedly increased by knockdown of GnRH-R expression [42]. In ovarian cancer cell lines EFO-21 and EFO-27 that express GnRH and GnRH-R, treatment with GnRH-agonist triptorelin (100 nM) resulted in an increase of cells in the G_0/1_ phase of the cell cycle and a decrease in the G_2/s_ phase. DNA synthesis was decreased to 45% of the control, likely meditated through JunD and AP-1 [43].

Takagi et al. demonstrated the presence of Gαi and Gαs proteins and the activation of phosphatase activity in plasma membranes from EC samples expressing GnRH-R [44]. Imai et al. showed that GnRH agonist induced protein dephosphorylation in plasma membranes from EC samples expressing GnRH-R. This effect was dose-dependently and completely inhibited by pertussis toxin [45]. Later, this group provided evidence that the GnRH-agonist leuprorelin stimulated intra-tumoral expression of the apoptosis-inducing Fas ligand in GnRH-R bearing EC. At 10 µM concentration, the GnRH agonist induced up to 90% reduction in the cell number preceded by Fas ligand production [46]. In HEC-1A EC cells and 36 human EC samples expressing GnRH-R mRNA, telomerase activity was not significantly altered by GnRH-agonist treatment. The expression of mRNA of human telomerase transferase, however, was markedly decreased [20]. Chatzaki and colleagues found no activation of GnRH-signal transduction mechanisms, known from pituitary gonadotropes including total inositol phosphate increase, cyclic AMP-production, and cytosolic Ca^2+^ after GnRH or GnRH agonist treatment of Ishikawa and HEC-1A cell lines and EC tissues [12]. Shibata et al. found that inhibition of proliferation of the HHUA EC cell line by the GnRH-agonist was mediated through activation of protein kinase C and subsequent increase of annexin V, which is a Ca^2+^-dependent phospholipid binding protein [13]. Borri et al. showed clear anti-proliferative effects of GnRH agonist leuprorelin and GnRH antagonist antide in human EC cell lines HEC-1, inhibitory effects on [^3^H] thymidine incorporation, and a slight accumulation of cells in the Go/G1 phase, but no effect on intracellular free calcium concentration [14]. Ohta et al. showed that GnRH-a inhibited telomerase activity but not mRNA expression for telomerase in six human EC cell lines [15]. Kim and colleagues demonstrated a cell cycle arrest at the G_1_-S transition of CUME-1 EC cells after treatment with GnRH agonist triptorelin [16]. Sica and coworkers found that two GnRH-agonists counteracted or even suppressed estrogen stimulated growth of Ishikawa cells and prevented estrogen-induced decrease in the level of estrogen receptors [33].

Park et al. showed that GnRH treatment induces integrin β3 expression and activation of focal adhesion kinase (FAK) and an activation of ERK 1/2 and p38MAPK [35]. Zhao et al. demonstrated that GnRH-agonist triptorelin inhibits proliferation of HEC-1A cells and induced apoptosis in a dose-dependent manner. This effect was augmented, when phosphatase and tensin homolog gene (*PTEN*) was knocked down [34]. Öztürk et al. found anti-proliferative effects of GnRH agonist leuprorelin and GnRH antagonist ganirelix in three primary EC cell lines associated with an increase of apoptotic cells [47].

The GnRH metabolite GnRH-(1-5), resulting from cleavage of the native decapeptide at the Tyr^5^-Gly^6^ bond, has autonomous mechanisms of action including stimulation of proliferation of the Ishikawa EC cell line by increasing EGF release and the phosphorylation of EGF receptors. This effect is likely mediated through the orphan G protein-coupled receptor 101 (GPR 101) [48,49]. Using the peptidomimetic GnRH-antagonist AEZS-115, Engel et al. could show a marked time-dependent and dose-dependent inhibition of human EC cell lines HEC-1A and Ishikawa growth that expressed mRNA for the GnRH-R. They concluded, however, that the antitumor activity of AEZS-115 was not mediated through the GnRH-R in the tumor cells [50].

### 3.5. GnRH-II and GnRH-II Receptors in EC

Apart from the well-known decapeptide GnRH-I, a second form, GnRH-II, was found to be universally present and preserved from jawed fish to humans [51]. Millar et al. cloned a type II GnRH-R from the marmoset monkey, which is highly specific for GnRH-II and found to be expressed in human tissues [52]. Neill et al. cloned the GnRH-II-R from the rhesus monkey [53]. The type II GnRH-R has a C-terminal and, in contrast to the type I GnRH-R, is rapidly internalized. In addition, GnRH-I antagonists had agonistic effects on the type II receptor [17]. We detected the expression of the mRNA for GnRH-II-R in HEC-1A and Ishikawa human EC cell lines using reverse transcriptase-polymerase chain reaction (RT-PCR) and Southern blot analysis. The proliferation of these cells was dose-dependently and time-dependently reduced by native GnRH-II, which was significantly more effective than GnRH-I agonist triptorelin. In the ovarian cancer cell line SK-OV3, that expresses GnRH-II-R but not GnRH-I-R, native GnRH-II but not GnRH-I agonist triptorelin had anti-proliferative effects [54]. GnRH agonist triptorelin, GnRH-antagonist cetrorelix, and native GnRH-II had anti-proliferative effects on human EC cell lines Ishikawa, HEC-1A, and HEC-1B that express receptors for GnRH-I and mRNA for receptors for GnRH-II. When the receptor for GnRH-1 was knocked out, the anti-proliferative effect of triptorelin was abrogated, while those of cetrorelix and GnRH-II persisted [55]. Since a functional human GnRH-II-R transcript has not been found so far, the mRNA detected by us is suspected to be non-functional because of a stop-codon in the sequence [24]. Using a specific anti-serum to human GnRH-II-R and Western blot analysis, we detected the expected 54-kDa band in ovaries from the marmoset monkey, where the expression of a GnRH-II-R had been shown. In human EC cell lines, Ishikawa and HEC-1A, as well as in human placenta, we obtained a 43-kDa band, which might represent a truncated, but functional 5 transmembrane domain human GnRH-II-R. Using a photo affinity labelling technique, with a ^125^I labelled GnRH-II analog, we obtained a band of 43 kDa. GnRH-I antagonist cetrorelix and GnRH-II but not GnRH-I agonist triptorelin were able to displace this binding [56]. In the human EC cell line HEC-1A, EGF induced autophosphorylation of the EGF-receptor, activation of MAP-kinase, and expression of the immediate early gene c-fos were inhibited by treatment with the GnRH-II agonist [D-Lys^6^] GnRH-II. After knock out of the GnRH-I-R expression, these effects of [D-Lys^6^] GnRH-II persisted [57].

Neither GnRH-I and GnRH-I-agonists nor GnRH-I-antagonists induced apoptosis in endometrial cancer cell lines. Antagonists of GnRH-II, however, resulted in apoptotic cell death via dose-dependent activation of caspase-3. In nude mice, GnRH-II markedly inhibited growth of xenotransplants of the human EC cell line HEC-1B [58]. GnRH-II antagonists were shown to activate apoptosis in human endometrial cancer cells through GnRH-I-R via activation of stress-induced mitogen-activated protein kinases p38 and c-Jun NH_2_ terminal kinase, leading to activation of pro-apoptotic protein Bax [59].

Kim and colleagues found that GnRH-II stimulates stress activated p38MAPK in an ovarian cancer cell line OVCAR-3, leading to an inhibition of proliferations and an increase of apoptosis [60].

Wu et al. inhibited proliferation and induced apoptosis in the human EC cell line Ishikawa by treatment with GnRH-II and provided evidence that this effect is mediated through the GnRH-I-R, activation of the ERK1/2 pathways, and induction of GADD45α-signalling [26]. Park et al. found that the inhibition of growth in the human EC cell line HEC-1A achieved by treatment with GnRH-I or GnRH-II is mediated through activation of integrin beta-3, focal adhesion kinase (FAK), and ERK1/2 and p38MAPK pathways. The effects of GnRH-II were more pronounced than those of GnRH-I [35]. Wu et al. found that, in Ishikawa and ECC-1 human EC cell lines, a GnRH-II agonist promoted cell motility through the GnRH-I-R and subsequent phosphorylation of ERK1/2 and JNK leading to activation of matrix metalloproteinase (MMP)-2 [27].

### 3.6. GnRH Receptor in EC as a Target for Cytotoxic Therapy

Apart from pituitary gonadotropes and reproductive organs, most other tissues and hematopoietic stem cells do not express the GnRH-R [61]. In almost all cases of EC, ovaries, fallopian tubes, and the uterus are surgically removed during primary treatment. The GnRH-R in tumor residues or metastases of EC could, therefore, be used to deliver a targeted therapy with improved anti-tumor efficacy and fewer side effects [61,62]. A number of GnRH-analogs covalently linked to a cytotoxic agent have been developed that bind specifically and with high affinity to GnRH-R in tumors including EC [61,62,63]. After internalization of the ligand receptor complex, the cytotoxic moiety is liberated and acts within the tumor cells [61,62,63]. We could show that the cytotoxic GnRH-agonist Zoptarelin Doxorubicin (AEZS-108, AN-152), in which the GnRH-agonist [D-Lys^6^] GnRH is covalently coupled to the cytotoxic agent doxorubicin selectively binds to GnRH-R in human EC cell lines. This is internalized and results in accumulation of doxorubicin in the nuclei of these cells. No binding and uptake of Zoptarelin Doxorubicin was detected in cell lines that did not express GnRH-R or in the presence of an excess of a GnRH-agonist competitively blocking the receptors [63]. Proliferation of cells was more potently inhibited by Zoptarelin Doxorubicin than by equimolar concentrations of free doxorubicin in cell lines expressing GnRH-R [63]. In nude mice, bearing xenografts of human EC cell line HEC-1B, tumor volumes were significantly reduced by treatment with Zoptarelin Doxorubicin, while treatment with equimolar concentrations of free doxorubicin only arrested tumor growth, but did not reduce their volume. No toxic side effects were observed with Zoptarelin Doxorubicin treatment, while free doxorubicin caused loss in body weight and death in the animals. The growth of tumors not expressing GnRH-R was not affected by treatment with Zoptarelin Doxorubicin [61]. In human EC cell lines, HEC-1A and Ishikawa Zoptarelin Doxorubicin induced apoptosis significantly more potently than doxorubicin without activating the multi-drug resistance 1(MDR-1) system [64].

Nechushtan et al. constructed a chimeric toxin based on GnRH and Pseudomonas exotoxin that bound specifically to human EC cell primary cultures and induced marked cell death [65]. Palyi and colleagues induced apoptosis in Ishikawa cells by treatment with conjugates of GnRH III-analogs and a copolymer [66]. Engel et al. showed that HEC-1A and RL-95-2 human EC cell lines express functional GnRH-R. Athymic mice bearing xenografts of these cell lines were treated with Zoptarelin Doxorubicin or another cytotoxic GnRH-agonist (AN-207). Tumor growth was significantly suppressed. The cytotoxic radicals (doxorubicin or 2-pyrrolinodoxorubicin) had no effects [22]. Yang et al. demonstrated that a conjugate of [D-Lys^6^] GnRH-Pro^9^-ethymamide with Pokeweed antiviral protein (PAP) was cytotoxic to the EC cell line Ishikawa. PAP alone, that cannot enter cells, had no effects. The cytotoxic activity of the GnRH-PAP conjugate was inhibited by an excess of a GnRH-agonist [21].

Li and colleagues developed a GnRH analog LHRH’ that binds with high affinity to GnRH-R but does not interfere with gonadotropin secretion. LHRH’ coupled to cecropin B, which is an antimicrobial peptide, significantly inhibited cell viability of EC cells HEC-1A but not of normal eukaryotic cells [67].

Our group developed a GnRH-R targeted gene therapy system for gynecological tumors, including EC that worked in vitro and in athymic mice bearing xenografts of HEC-1B human RC cells [68].

### 3.7. Clinical Applications of GnRH-Analogs in EC and Atypical Endometrial Hyperplasia (AEH)

GnRH-agonists and antagonists have been used in the treatment of EC and its precancer AEH based on two rationales: 1. Suppression of pituitary gonadotropin secretion leading to suppression of ovarian function including estrogen and progesterone production, which is a state called reversible medical castration [6]. This strategy has been applied for the conservative therapy of young women with AEH and early EC wishing to preserve their fertility. 2. Exploitation of the direct anti-tumor effects of GnRH-analogs on GnRH-R on the tumor cells. A third approach has been the application of cytotoxic GnRH-analogs for targeted chemotherapy of tumors expressing GnRH-R.

### 3.8. Suppression of Ovarian Steroid Production by GnRH-Analogs as a Treatment for AEH and Early EC

Pérez-Medina et al. treated 10 patients with AEH with the progestogen norethisterone acetate for three months and monthly injections of Triptorelin for six months. At a five-year follow up, regression was observed in 84.2% of patients, persistence in 5% of patients, recurrence in 5% of patients, and progression in 5% of patients [69]. Minig et al. treated 20 patients with AEH and 14 with early EC with a levonorgestrel-intrauterine device (LNG-IUD) for one year plus the GnRH-analog for six months. They observed a complete response in 95% of patients with AEH and 57.1% in women with EC G1. Progression was observed in 5% (AEH) and in 28% (EC) of patients, respectively. Four of 20 patients with AEH and 2 of 24 with early EC had recurrences. Nine women had 11 spontaneous pregnancies [70]. Pashov and colleagues treated 13 women with AEH with leuprorelin acetate (six monthly injections) and an LNG-IUD Eleven women with stage IA well differentiated EC were treated with nine injections of GnRH and a LNG-IUD for at least 12 months [71]. They reported that the therapy was effective for all patients. Three women with EC became pregnant. Pronin et al., prospectively, treated 32 women with G1 EC with LNG-IUD and a GnRH agonist for at least six months. Twenty-three (72%) of the patients had a complete remission (CR). Two had a recurrence after the complete response [72]. Zhou et al. treated 29 patients with well-differentiated EC or AEH with monthly injections of a depot GnRH agonist combined with a LNG-IUD or the aromatase inhibitor letrozole (2.5 mg/day). 88.2% of the EC patients and 12 women (100%) in the AEH group had a complete response. 8% with AEH and 6% of women with EC had recurrence after CR [73]. Zhang et al. treated six young obese patients with EC with monthly injections of depot GnRH-agonist and aromatase inhibitor. The CR rate was 100%. After a median follow-up of four years, no recurrence was observed. The pregnancy rate was 50% and the life birth rate was 75% [74]. Tock et al., retrospectively, analyzed 18 women younger than 41 years with grade 1 EC (39%) or AEH (50%), who received endometrial resection and, subsequently, a GnRH agonist for three months. They observed a CR in 12 patients (67%) and three relapses (25%) after CR. Eight women had 14 pregnancies [75].

Analyzing the data from 127 patients with AEH (75%) or early EC (25%) after different fertility sparing treatments, Yin et al. found that the treatment with a GnRH agonist with or without an LNG-IUD/aromatase inhibitor might be preferable for obese patients, resulting in fewer relapses after primary CR [76] (Table 1).

### 3.9. Clinical Studies on Direct Anti-Tumor Effects of GnRH-Analogs in EC 

Gallagher et al. treated 17 patients with EC that had recurred after surgery, radiotherapy, and progesterone treatment with monthly injections of a GnRH agonist, achieving an objective remission in six patients (35%). This continued for a median of 20 months with no adverse effects [77]. Jeyarajah et al. reported on these 17 patients and an additional 15 patients with recurrent EC with progressive, symptomatic, and measurable disease, treated with monthly injections of Leuprorelin or Goserelin. An objective response was obtained in 9 of 32 patients (28%) including pelvic as well as distant sites of recurrence. The response rate did not correlate with differentiation of the tumors (15 patients with G3 EC). Duration of responses was 4 to 86 months [78].

Covens et al. performed a phase II trial on 25 patients with recurrent or metastatic EC. Treatment with GnRH agonist Leuprorelin (7.5 mg i. m. every 28 days resulted in no objective response. Eight patients had stable disease for a median of 5 months, 14 patients had progressive disease, and three patients were not evaluable [79]. Lhommé et al. treated 24 eligible patients with advanced or recurrent EC with monthly injections of the GnRH agonist triptorelin depot. They observed one complete and one partial response (response rate 8.7%) and five disease stabilizations, often of long duration. Toxicity was very low [80]. Noci et al. reported on a patient with EC where a surgical treatment was not possible due to comorbidities. The tumor expressed mRNA for GnRH-R. Under treatment with leuprorelin, she remained stable for six years [81]. Asbury et al. treated 40 patients with advanced or recurrent EC with monthly s.c. implants of the GnRH agonist goserelin. They observed two complete and three partial remissions (12%). No severe or life threatening toxicities occurred because of the GnRH agonist [82]. (Table 2)

### 3.10. Clinical Studies on Cytotoxic GnRH-Analogs in EC

In a phase I study, we determined the maximal tolerated dose of Zoptarelin Doxorubicin and characterized dose limiting toxicities in 17 women with ovarian, endometrial, or breast cancers for which standard curative or palliative measures could not be used. In each patient, the primary tumor or the metastatic lesion expressed GnRH-R as determined by immunochemistry. The maximal tolerated dose of Zoptarelin Doxorubicin in the absence of supportive medication was 267 mg/m^2^, equimolar to 76.8 mg/m^2^ of free doxorubicin. One EC patient in the 160 mg/m^2^ and 267 mg/m^2^ groups each, which were considered to be therapeutically effective, had a complete remission. Toxicity was lower for Zoptarelin/Doxorubicin than for free doxorubicin. Pituitary gonadotropin secretion was partly suppressed but other pituitary cells were not influenced [83]. In a subsequent Phase II trial, 43 eligible patients with advanced or recurrent EC that expressed GnRH-R were treated with Zoptarelin Doxorubicin (267 mg/m^2^, 2-h infusion every 21 days). Two patients (5%) had a complete remission and eight patients (18%) had a partial remission. Stable disease for at least six weeks was observed in 44% of patients, resulting in a clinical benefit rate of 67%. There was no difference in response between patients with type 1 or type 2 EC. The most frequently reported grade 3 or 4 adverse effects were neutropenia (12%) and leucopenia (9%) [25]. Based on these results, a phase III trial on 511 patients with advanced, recurrent, or metastatic EC who had failed to prior platinum and Taxane therapy was performed (ClinicalTrials.gov Identifier: NCT01767155). GnRH-R status was not determined. Patients were randomized to obtain Zoptarelin Doxorubicin (267 mg/m^2^) or doxorubicin (60 mg/m^2^) every 21 days for up to nine cycles. The median OS for Zoptarelin Doxorubicin was 10.9 months compared to 10.8 months for patients treated with doxorubicin.Progression free survival (PFS) was 4.7 months for both groups. The objective response rate was 12% vs. 14% and the clinical benefit rate was 54% vs. 52%. Side effects were not different between the two treatments [84,85].

## 4. Discussion

### 4.1. GnRH-Receptors in EC

The data reviewed above clearly suggest that most human EC cell lines and primary EC express high affinity/low-capacity receptors for GnRH. It is true that some groups were not able to find these GnRH-R [10,12,14]. However, most researchers worldwide detected them either by binding assay, RT-PCR, restriction enzyme analysisand Southern blot analysis or immunohistochemistry [7,9,11,13,15,16,17,19,20,21,22,23,24,25,26,27,28]. The sequence of the cDNA of the GnRH-R in HEC-1A and Ishikawa human EC cell lines is identical to that of the GnRH-R in a human pituitary gland [18]. Approximately 50–95% of primary human EC express GnRH-R [7,11,17,19,20,23,24,25,28].

### 4.2. Expression of GnRH in EC

The production of GnRH by EC-cell lines and in 75–100% of primary tumor samples was shown by immunoassay, bioassay, high performance liquid chromatography, immunohistochemistry, RT-PCR, restriction enzyme analysis, and Southern blot analysis [12,24,29,30], so that this finding is also well accepted.

### 4.3. Effects of GnRH, GnRH-Agonists, and GnRH Antagonists on the Proliferation of EC Cells

In established human EC cell lines as well as in primary cultures of EC, most researchers found a growth inhibition induced by treatment with GnRH-agonists [9,13,14,15,17,20,34,35]. Comparable anti-proliferative effects were seen, when GnRH-antagonists were used [9,14,17,31]. Under serum free conditions, IGF-1, EGF, and estrogen induced proliferation of EC cell lines was reduced by GnRH analogs [31,32,33]. Significant anti-proliferative effects of GnRH analogs were seen at 10 pM to 1 nM concentrations [9], but relevant growth inhibition was observed at higher concentrations (1 µM, 10 µM) [9,13,14,15,17,20,32,33,34,35].

A common finding made by all groups was that GnRH-antagonists had the same anti-proliferative effects on EC cells as GnRH-agonists, suggesting that the dichotomy between GnRH-agonists and antagonists, known from the pituitary, is not valid in EC cells [6]. To elucidate the effects of GnRH produced and secreted by the tumor cells, we treated cell cultures of human ovarian cancer cell lines EFO-21 and EFO-27 that had been shown to express high affinity GnRH-R and to secrete GnRH with neutralizing concentrations of antiserum to GnRH. This resulted in a significant stimulation of proliferation. Native GnRH in low concentrations had no or little anti-proliferative effect [36].

Thus, it seems reasonable to conclude that the majority of human EC express GnRH and GnRH-R as an autocrine system reducing their proliferation. Both GnRH agonists and antagonists have dose-dependent anti-proliferative effects.

### 4.4. Signal Transduction and Intracellular Actions of GnRH in EC

In the pituitary gonadotrope GnRH-R that have bound the GnRH couple to G-protein αq and induce activation of phospholipase (PLC), protein kinase (PKC), and adenylyl cyclase (AC) [12,14,32]. These enzymes and pathways are present in human EC cell lines but are not involved in the mediation of anti-proliferative effects of GnRH agonists [32]. Upon binding of a GnRH agonist or antagonist, the GnRH-R rather couples to G-protein αi and activates a phosphotyrosine phosphatase that reduces EGF induced tyrosine phosphorylation of the EGF-R [18,32,38,44,45,46]. This results in a suppression of the Ras/MAPK/ERK pathway and an inhibition of c-fos expression [18,32,38] (Figure 1). This mechanism has been shown in human breast and ovarian cancer cell lines [86]. In addition, through this mechanism, GnRH-agonists can inhibit E_2_ induced cell proliferation of ERα-positive human endometrial, ovarian, and breast cancer cell lines (Figure 1) [40]. In breast cancers, signalling of membrane-bound G-Protein-coupled estrogen receptor 1 (GPER) through transactivation of EGF-R could also be inhibited by GnRH-agonist treatment (Figure 1) [86]. Thus, E_2_-induced proliferation of ERα-negative but GPER-positive breast cancer cells could be prevented by treatment with GnRH agonists.

GnRH agonists stimulated via G-protein αi the JNK/AP-1 pathway, leading to inhibition of the cell cycle (Figure 1) [39]. Native GnRH, GnRH-agonists, and GnRH-antagonists did not induce apoptosis but rather protected endometrial cancer cells from apoptosis induction by UV-light or the cytotoxic agent doxorubicin through activation of NF-κB (Figure 1) [42]. Thus, we conclude from our data and those of others, that the expression of GnRH and GnRH-R in human EC is part of an autocrine system, counteracting the proliferative effects of growth factors and estrogens, and increasing AP-1 expression, leading to cell cycle arrest. In addition, this GnRH system reduces apoptosis (Figure 1). Other authors, however, found induction of apoptosis caused by GnRH-agonists and/or antagonists, the activation of protein kinase C, and other mechanisms including FasL and FAS, telomerase transferase, telomerase activity, and annexin V [13,15,16,20,34,35,46,47].

The reasons for these discrepancies are unknown. It is not unreasonable to speculate that the GnRH-R in tumor cells can couple to multiple signal-transduction pathways depending on the cellular milieu in different cell lines and even different passages of the same cell line [86].

### 4.5. GnRH-II and GnRH-II-Receptors in EC

**Figure 2:**
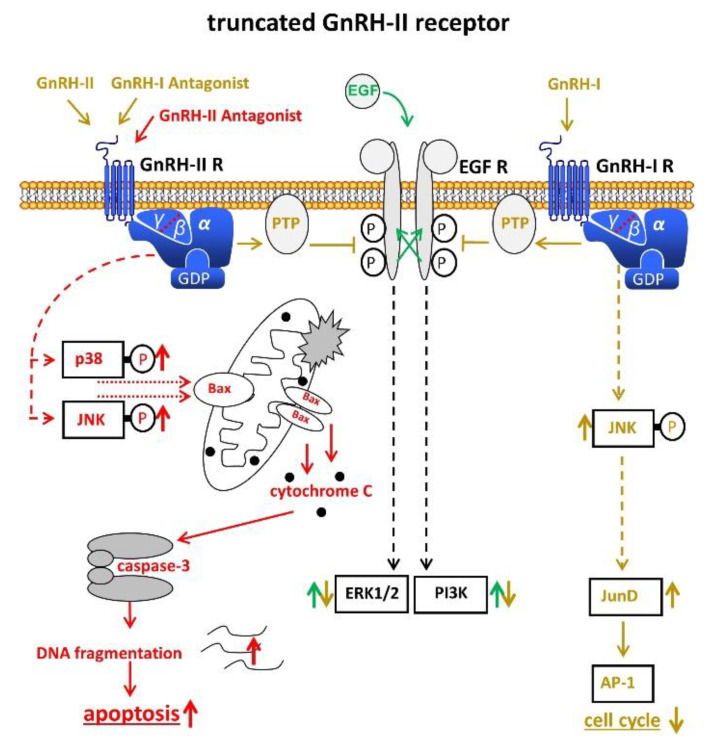
Signal transduction of the putative truncated GnRH-II receptor in endometrial cancer (EC). Binding of GnRH-II, GnRH-II agonists, and GnRH-I antagonists cause activation of PTP, leading to dephopsphorylation of activated EGF-R with the consequences described in Figure 1. Binding of GnRH-II antagonists induce apoptosis through activation of p38, JNK, and the intrinsic apoptotic pathway. AP-1, activvator protein-1. Bax, B-cell lymphoma 2 (Bcl-2)-associated X protein. EGF, epidermal growth factor. EGF-R, EGF receptor. ERK1/2, p44/42 mitogen-activated protein (MAP) kinase. GDP, guanosindiphosphat. GnRH, gonadotropin releasing hormone. JNK, c-Jun N-terminale kinase. JunD, transcription factor JunD. P38, mitogen-activated protein kinase P38. PI3K, phosphoinositide 3-kinase. PTP, protein tyrosine phosphatase.

The data from our group suggest that a truncated, but functional 5-transmembrane human GnRH-II-R, is expressed in human endometrial and ovarian cancers. After binding of the GnRH-antagonist, native GnRH-II, or GnRH-II-agonist, it couples to G protein αi and activates the signal transduction mechanisms described for the GnRH-I-R in human cancers including inhibition of autophosphorylation of the EGF-receptor and phosphorylation of JNK [54,55,57,58] (Figure 2). Our findings suggest that the antiproliferative effects of GnRH-I antagonists are mediated through GnRH-II-R. The anti-proliferative effects of native GnRH-II mediated through GnRH-II-R were much more pronounced than those of GnRH-I agonist mediated through GnRH-I-R [54,55,56,57,58].

GnRH-I, GnRH-I-agonists, GnRH-I-antagonists, and GnRH-II-agonists did not increase apoptosis in EC, ovarian, or breast cancer cell lines, but rather protected cells from programmed cell death [58,59]. Antagonists of GnRH-II, however, potently induced apoptosis in human endometrial, ovarian, and breast cancer cells, mediated through activation of stress-induced mitogen activated protein kinases p38 and c-JunNH2-terminal kinase, leading to activation of proapoptotic protein Bax [58,59] (Figure 2). GnRH-II antagonists potently inhibited the growth of human endometrial and ovarian cancers in nude mice [58].

**Figure 3:**
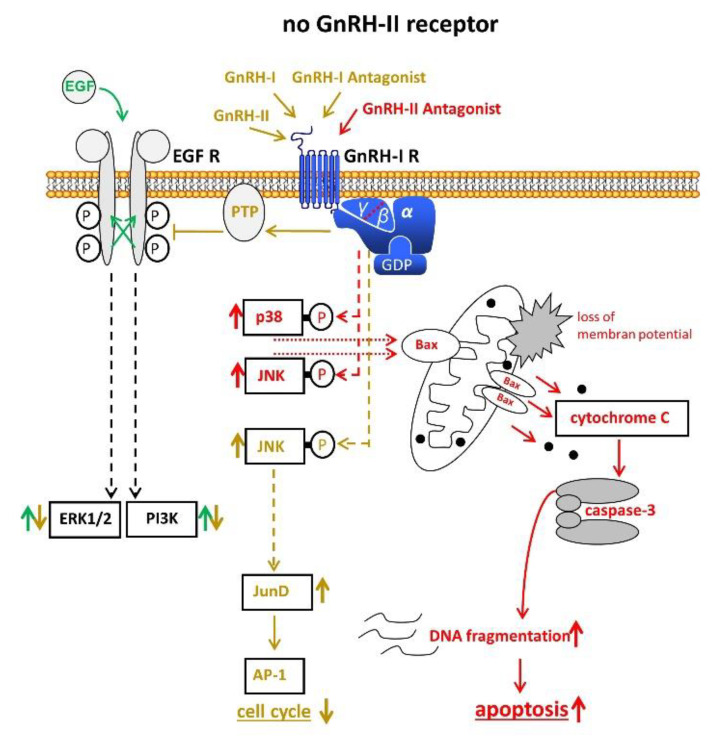
Signal transduction of GnRH-I and GnRH-II and their agonistic as well as antagonistic analogs through the GnRH-I-R if no functional GnRH-II-R is expressed. For details, cf. Figure 1 and Figure 2. AP-1, activvator protein-1. Bax, B-cell lymphoma 2 (Bcl-2)-associated X protein. EGF, epidermal growth factor. EGF-R, EGF receptor. ERK1/2, p44/42 mitogen-activated protein (MAP) kinase. GDP, guanosindiphosphat. GnRH, gonadotropin releasing hormone. JNK, c-Jun N-terminale kinase. JunD, transcription factor JunD. P38, mitogen-activated protein kinase P38. PI3K, phosphoinositide 3-kinase. PTP, protein tyrosine phosphatase.

Other groups provided evidence that no functional GnRH-II-R is expressed in the human and that the effects of GnRH-II are mediated through GnRH-I-R [26,27,35,39] (Figure 3).

### 4.6. GnRH-R in EC as a Target for Cytotoxic Therapy

The in vitro and in vivo experimental data available suggest that GnRH-R can be utilized as a target to specifically introduce toxic molecules into EC cells [21,61,62,63,64,65]. Of particular interest is the finding that cytotoxic GnRH-molecules can bypass the multi-drug resistance 1 system that eventually makes tumor cells refractory to chemotherapy [64].

### 4.7. Clinical Application of GnRH Analogs in EC and Atypical Endometrial Hyperplasia (AEH)

Estrogen withdrawal by the GnRH-agonist induced reversible medical castration, which has been used in combination with systemic or local progestogen application as a fertility sparing treatment for AEH and early EC [69,70,71,72,73,76]. Alternatively, GnRH agonists were combined with aromatase inhibitors for more intensive suppression of estrogens [73,74,76] or given alone after complete endometrial resection [75]. In these small series, complete response rates of about 70% (54–100%) were observed for early EC. Complete response rates were higher in AEH (80–100%). Pregnancy rates were about 50%. A relevant number of patients had progression during therapy or relapsed after complete remission [69,70,71,72,73,74,75,76]. Estrogen withdrawal through reversible medical castration by GnRH analogs alone or in combination with a LNG-IUD or an aromatase inhibitor seems to be preferable in obese patients [76] (Table 1).

A series of small phase II trials has assessed the efficacy of conventional doses of GnRH-agonists in the treatment of recurrent or advanced EC no longer amenable to surgery or radiotherapy, including patients with G3 tumors. Objective responses were observed in 0% to 35%, which tended to be long lasting. Toxicity was low [77,78,79,80,81,82] (Table 2). It has to be considered that the serum concentrations of GnRH-agonists achieved with standard depot-preparations are in the nanomolar range and sufficient to down-regulate pituitary GnRH-R [6]. For optimal direct effects of GnRH-I-agonists or antagonists on EC cells, much higher concentrations (µM) are needed [9,13,14,15,17,20,31,32,34,35]. Clinical trials with higher doses of GnRH-I-analogs have not been performed. GnRH-II analogs, especially GnRH-II antagonists that are much more potent in vitro and in vivo [58,59], should be tested in EC patients.

Phase I and II trials with the cytotoxic GnRH analog Zoptarelin Doxorubicin resulted in an objective response rate of 23% and a clinical benefit rate of 67% in patients with advanced or recurrent EC that expressed GnRH-R [25]. Toxicity was low. In a phase III trial with patients with recurrent or metastatic EC that had failed to prior platinum chemotherapy, an equipotency of Zoptarelin Doxorubicin and free Doxorubicin was found. However, the GnRH-R-status of these tumors was unknown. Considering that Zoptarelin Doxorubicin had been shown to be ineffective in EC not expressing GnRH-R [61,62,63] and that 5–50% of EC have no GnRH-R [20,23,24,25], it is reasonable to speculate that the Zoptarelin Doxorubicin is more efficacious in patients with EC that express GnRH-R.

## 5. Conclusions and Perspectives

Treatment with conventional doses of GnRH-agonists that suppress pituitary gonadotropin secretion and ovarian estrogen production has become part of fertility preserving therapy either alone or in combination with LNG-IUD (52 mg) or aromatase inhibitors in young patients with AEH or early EC (EEC).

There is convincing evidence that EC express GnRH-I-R and GnRH-I as a negative autocrine system, limiting cell proliferation and likely apoptosis. It is unclear whether a functional GnRH-II-R exist in humans. The GnRH-I-R and, if existent, the putative GnRH-II-R can be targeted by analogs of GnRH-I and GnRH-II to inhibit proliferation and to induce apoptosis. Clinical trials to exploit these direct anti-tumor effects have, so far, been performed with conventional doses of GnRH agonists, resulting in marginal efficacy, but low toxicity. Trials using higher doses of GnRH-I analogs or the more potent GnRH-II analogs still have to be performed. The cytotoxic GnRH-analog Zoptarelin Doxorubicin has been shown to have meaningful activity in animal models and a phase II trial. A well-designed phase III trial with patients with advanced or recurrent EC expressing GnRH-R is warranted.

## Figures and Tables

**Table 1:** Suppression of ovarian estrogen production through GnRH-analogs as treatment for atypical endometrial hyperplasia (AEH) and early endometrial cancer (EEC).

First Author, Year (Reference)	Recruitment Phase	Patient Age	Study Design	Diagnosis (Number of Evaluable Patients)	Intervention	Results
Perez-Medina, 1999 [69]	1991–1993	29–65 years (mean: 48 y)	prospective cohort	AEH (*n* = 19)	Triptorelin-depot 3.75 mg i.m./month for 6 months plus NETA 500 mg i.m./week for 3 months	after 5 years: 84.2% regression 5.1% persistence 5.1% recurrence 5.1% progression
Minig, 2011 [70]	1996–2009	22–40 years (mean: 34 y)	prospective cohort	AEH (*n* = 20) EEC (*n* = 14)	Triptorelin-depot 3.75 mg i.m./month for 6 months LNG-IUD (52 mg) for 1 year	after 4–102 months: AEH: 95% CR 5% progression 20% recurrence EEC: 57.1% CR 28% progression 14% recurrence 11 pregnancies
Pashov, 2012 [71]	2006–2010	23–35 years (mean: 29 y)	prospective cohort	AEH (*n* = 13) EEC (*n* = 11)	Leuprorelin-depot 3.75 mg i.m./month (AEH 6 months), EEC 9 months) LNG-IUD (52 mg) for 1 year	after 2–6 years: AEH: 100% CR EEC: 100% CR 3 pregnancies
Pronin, 2015 [72]	2009–2012	<42 years (mean: 33 y)	prospective cohort	EEC (*n* = 32)	Goserelin-depot 3.6 mg s.c./month for 6 to 12 months LNG-IUD (52 mg) for 6 to 12 months	after 1–2 years: 72% CR 21% persistence 6% recurrence
Zhou, 2017 [73]	2012–2016	21–42 years (mean: 30.6 y)	retrospective cohort	AEH (*n* = 12) EEC (*n* = 17)	GnRH-agonist (no further details) 3.75 mg i.m./month for ≥3 months plus LNG-IUD (52 mg) for ≥6 months or letrozole (2.5 mg p.o./day) for ≥3 months	after 5.6–54.9 months: AEH: 100% CR 8.3% recurrence EEC: 88% CR 5.9% PR 5.9% SD 5.9% recurrence
Zhang, 2019 [74]	2010–2015	mean age: 30.5 ± 3.3 y	prospective cohort	EEC (*n* = 6)	Triptorelin-depot 3.75 mg i.m./month for 6 to 9 months plus letrozole (2.5 mg p.o./day) for 6 to 9 months	after 1.3–7 years: 100% CR no recurrence pregnancy rate: 50%
Tock, 2018 [75]	1999–2016	18–41 y	Retrospective case series	AEH (*n* = 9) EEC (*n* = 9)	endometrial resection pus 3.6 mg Goserelin-depot s.c./month for 3 months	after 5–180 months: AEH: 78% CR 22% SD 22% recurrence EEC: 56% CR 44% SD 11% recurrence 14 pregnancies

Abbreviations: AEH, atypical endometrial hyperplasia. EEC, early endometrial cancer. CR, complete remission. PR, partial remission. SD, stable disease. NETA, norethisterone acetate. LNG-IUD, levonorgestrel-intrauterine device. i.m., intramuscular. s.c., subcutaneous. p.o., per os.

**Table 2:** Effects of GnRH-agonists (conventional doses) in patients with advanced or recurrent endometrial cancer (EC).

First Author, Year (Reference)	Recruitment Phase	Patient Age	Study Design	Diagnosis (Number of Evaluable Patients)	Intervention	Results
Jeyarajah, 1996 [78] including 17 pts from [77]	not indicated	34–88 years	prospective cohort (2 centers)	recurrent EC, progression onconventional treatment (*n* = 32) G 1/2 (*n* = 16) high grade (*n* = 16)	Leuprorelin-depot 3.75–7.5 mg i.m./month (31 pts) Goserelin-depot s.c. 3.6 mg/month	28% OR duration: 4–86 months no difference between G3 and G1/2 tumors
Covens, 1997 [79]	1993–1995	42–75 years	Multicenter phase II study	recurrent or metastatic EC (*n* = 25)	Leuprorelin-depot 7.5 mg i.m./month	0% OR 33% SD (1–8 months)
Lhomme, 1999 [80]	1992–1994	46–85 years	Multicenter phase II study	recurrent or advanced EC (*n* = 23)	Triptorelin-depot 3.75 mg i.m./month	8.7% OR 21.7% SD (4–12 months)
Asbury, 2002 [82]	1996	median age: 71 y	multicenter phase II study	recurrent EC (*n* = 40)	Goserelin-depot 3.6 mg s.c./month	11% OR

Abbreviations: OR, objective responses. SD, stable disease. i.m., intramuscular. s.c., subcutaneous.

## Data Availability

Not applicable.

## References

[1] International Agency for Research on Cancer Cancer Today. Corpus Uteri. https://gco.iarc.fr/today/factsheets-cancers.

[2] Morice P., Leary A., Creutzberg C., Abu-Rustum N., Darai E. (2016). Endometrial cancer. Lancet.

[3] Ignatov A., Ortmann O. (2020). Endocrine risk factors of endometrial cancer: Polycystic ovary syndrome, oral contraceptives, infertility, tamoxifen. Cancers.

[4] Tempfer C.B., Hilal Z., Kern P., Juhasz-Boess I., Rezniczek G.A. (2020). Menopausal hormone therapy and risk of endometrial cancer: A systematic review. Cancers.

[5] Emons G., Mustea A., Tempfer C. (2020). Tamoxifen and endometrial cancer: A janus-headed drug. Cancers.

[6] Emons G., Schally A.V. (1994). The use of luteinizing hormone releasing hormone agonists and antagonists in gynaecological cancers. Hum. Reprod..

[7] Srkalovic G., Wittliff J.L., Schally A.V. (1990). Detection and partial characterization of receptors for [D-Trp6]-luteinizing hormone-releasing hormone and epidermal growth factor in human endometrial carcinoma. Cancer Res..

[8] Pahwa G.S., Kullander S., Vollmer G., Oberheuser F., Knuppen R., Emons G. (1991). Specific low affinity binding sites for gonadotropin-releasing hormone in human endometrial carcinomata. Eur. J. Obs. Gynecol. Reprod. Biol..

[9] Emons G., Schroder B., Ortmann O., Westphalen S., Schulz K.D., Schally A.V. (1993). High affinity binding and direct antiproliferative effects of luteinizing hormone-releasing hormone analogs in human endometrial cancer cell lines. J. Clin. Endocrinol. Metab..

[10] Peterson C.M., Jolles C.J., Carrell D.T., Straight R.C., Jones K.P., Poulson A.M., Hatasaka H.H. (1994). GnRH agonist therapy in human ovarian epithelial carcinoma (OVCAR-3) heterotransplanted in the nude mouse is characterized by latency and transience. Gynecol. Oncol..

[11] Imai A., Ohno T., Iida K., Fuseya T., Furui T., Tamaya T. (1994). Presence of gonadotropin-releasing hormone receptor and its messenger ribonucleic acid in endometrial carcinoma and endometrium. Gynecol. Oncol..

[12] Chatzaki E., Bax C.M., Eidne K.A., Anderson L., Grudzinskas J.G., Gallagher C.J. (1996). The expression of gonadotropin-releasing hormone and its receptor in endometrial cancer, and its relevance as an autocrine growth factor. Cancer Res..

[13] Shibata S., Sato H., Ota H., Karube A., Takahashi O., Tanaka T. (1997). Involvement of annexin V in antiproliferative effects of gonadotropin-releasing hormone agonists on human endometrial cancer cell line. Gynecol. Oncol..

[14] Borri P., Coronnello M., Noci I., Pesciullesi A., Peri A., Caligiani R., Maggi M., Torricelli F., Scarselli G., Chieffi O. (1998). Differential inhibitory effects on human endometrial carcinoma cell growth of luteinizing hormone-releasing hormone analogues. Gynecol. Oncol..

[15] Ohta H., Sakamoto H., Satoh K. (1998). In vitro effects of gonadotropin-releasing hormone (GnRH) analogue on cancer cell sensitivity to cis-platinum. Cancer Lett..

[16] Kim J.W., Lee Y.S., Kim B.K., Park D.C., Lee J.M., Kim I.K., Namkoong S.E. (1999). Cell cycle arrest in endometrial carcinoma cells exposed to gonadotropin-releasing hormone analog. Gynecol. Oncol..

[17] Noci I., Coronnello M., Borri P., Borrani E., Giachi M., Chieffi O., Marchionni M., Paglierani M., Buccoliero A.M., Cherubini A. (2000). Inhibitory effect of luteinising hormone-releasing hormone analogues on human endometrial cancer in vitro. Cancer Lett..

[18] Gründker C., Völker P., Emons G. (2001). Antiproliferative signaling of luteinizing hormone-releasing hormone in human endometrial and ovarian cancer cells through G protein alpha(I)-mediated activation of phosphotyrosine phosphatase. Endocrinology.

[19] Volker P., Grundker C., Schmidt O., Schulz K.D., Emons G. (2002). Expression of receptors for luteinizing hormone-releasing hormone in human ovarian and endometrial cancers: Frequency, autoregulation, and correlation with direct antiproliferative activity of luteinizing hormone-releasing hormone analogues. Am. J. Obs. Gynecol..

[20] Nagai N., Oshita T., Mukai K., Shiroyama Y., Shigemasa K., Ohama K. (2002). GnRH agonist inhibits human telomerase reverse transcriptase mRNA expression in endometrial cancer cells. Int. J. Mol. Med..

[21] Yang W.H., Wieczorck M., Allen M.C., Nett T.M. (2003). Cytotoxic activity of gonadotropin-releasing hormone (GnRH)-pokeweed antiviral protein conjugates in cell lines expressing GnRH receptors. Endocrinology.

[22] Engel J.B., Keller G., Schally A.V., Nagy A., Chism D.D., Halmos G. (2005). Effective treatment of experimental human endometrial cancers with targeted cytotoxic luteinizing hormone-releasing hormone analogues AN-152 and AN-207. Fertil. Steril..

[23] Jeon Y.T., Kim Y.B., Park S.Y., Kim J.W., Park N.H., Kang S.B., Song Y.S. (2009). Gonadotropin-releasing hormone receptor expression in endometrial cancer. Int. J. Gynecol. Pathol..

[24] Jankowska A.G., Andrusiewicz M., Fischer N., Warchol P.J. (2010). Expression of hCG and GnRHs and their receptors in endometrial carcinoma and hyperplasia. Int. J. Gynecol. Cancer.

[25] Emons G., Gorchev G., Harter P., Wimberger P., Stahle A., Hanker L., Hilpert F., Beckmann M.W., Dall P., Grundker C. (2014). Efficacy and safety of AEZS-108 (LHRH agonist linked to doxorubicin) in women with advanced or recurrent endometrial cancer expressing LHRH receptors: A multicenter phase 2 trial (AGO-GYN5). Int. J. Gynecol. Cancer.

[26] Wu H.M., Cheng J.C., Wang H.S., Huang H.Y., MacCalman C.D., Leung P.C. (2009). Gonadotropin-releasing hormone type II induces apoptosis of human endometrial cancer cells by activating GADD45alpha. Cancer Res..

[27] Wu H.M., Wang H.S., Huang H.Y., Lai C.H., Lee C.L., Soong Y.K., Leung P.C. (2013). Gonadotropin-releasing hormone type II (GnRH-II) agonist regulates the invasiveness of endometrial cancer cells through the GnRH-I receptor and mitogen-activated protein kinase (MAPK)-dependent activation of matrix metalloproteinase (MMP)-2. BMC Cancer.

[28] Hao D., Sun L., Hu X., Hao X. (2017). (99m)Tc-LHRH in tumor receptor imaging. Oncol. Lett..

[29] Irmer G., Burger C., Ortmann O., Schulz K.D., Emons G. (1994). Expression of luteinizing hormone releasing hormone and its mRNA in human endometrial cancer cell lines. J. Clin. Endocrinol. Metab..

[30] Furui T., Imai A., Tamaya T. (2002). Intratumoral level of gonadotropin-releasing hormone in ovarian and endometrial cancers. Oncol. Rep..

[31] Kleinman D., Roberts C.T., LeRoith D., Schally A.V., Levy J., Sharoni Y. (1993). Regulation of endometrial cancer cell growth by insulin-like growth factors and the luteinizing hormone-releasing hormone antagonist SB-75. Regul. Pept..

[32] Emons G., Muller V., Ortmann O., Grossmann G., Trautner U., Stuckrad B., Schulz K., Schally A. (1996). Luteinizing hormone-releasing hormone agonist triptorelin antagonizes signal transduction and mitogenic activity of epidermal growth factor in human ovarian and endometrial cancer cell lines. Int. J. Oncol..

[33] Sica G., Schinzari G., Angelucci C., Lama G., Iacopino F. (2001). Direct effects of GnRH agonists in human hormone-sensitive endometrial cells. Mol. Cell. Endocrinol..

[34] Zhao L.J., Liu N., Li X.P., Wang J.L., Wei L.H. (2010). Phosphatase and tensin homolog gene inhibits the effect induced by gonadotropin-releasing hormone subtypes in human endometrial carcinoma cells. Chin. Med. J..

[35] Park D.W., Choi K.C., MacCalman C.D., Leung P.C. (2009). Gonadotropin-releasing hormone (GnRH)-I and GnRH-II induce cell growth inhibition in human endometrial cancer cells: Involvement of integrin beta3 and focal adhesion kinase. Reprod. Biol. Endocrinol..

[36] Emons G., Weiss S., Ortmann O., Grundker C., Schulz K.D. (2000). LHRH might act as a negative autocrine regulator of proliferation of human ovarian cancer. Eur. J. Endocrinol..

[37] Kleinman D., Douvdevani A., Schally A.V., Levy J., Sharoni Y. (1994). Direct growth inhibition of human endometrial cancer cells by the gonadotropin-releasing hormone antagonist SB-75: Role of apoptosis. Am. J. Obs. Gynecol..

[38] Gründker C., Völker P., Schulz K.D., Emons G. (2000). Luteinizing hormone-releasing hormone agonist triptorelin and antagonist cetrorelix inhibit EGF-induced c-fos expression in human gynecological cancers. Gynecol. Oncol..

[39] Grundker C., Schlotawa L., Viereck V., Emons G. (2001). Protein kinase C-independent stimulation of activator protein-1 and c-Jun N-terminal kinase activity in human endometrial cancer cells by the LHRH agonist triptorelin. Eur. J. Endocrinol..

[40] Grundker C., Gunthert A.R., Hellriegel M., Emons G. (2004). Gonadotropin-releasing hormone (GnRH) agonist triptorelin inhibits estradiol-induced serum response element (SRE) activation and c-fos expression in human endometrial, ovarian and breast cancer cells. Eur. J. Endocrinol..

[41] Gunthert A.R., Grundker C., Bottcher B., Emons G. (2004). Luteinizing hormone-releasing hormone (LHRH) inhibits apoptosis induced by cytotoxic agent and UV-light but not apoptosis mediated through CD95 in human ovarian and endometrial cancer cells. Anticancer Res..

[42] Fister S., Schlotawa L., Gunthert A.R., Emons G., Grundker C. (2008). Increase of doxorubicin-induced apoptosis after knock-down of gonadotropin-releasing hormone receptor expression in human endometrial, ovarian and breast cancer cells. Gynecol. Endocrinol..

[43] Günthert A.R., Gründker C., Hollmann K., Emons G. (2002). Luteinizing hormone-releasing hormone induces JunD-DNA binding and extends cell cycle in human ovarian cancer cells. Biochem. Biophys. Res. Commun..

[44] Takagi H., Imai A., Horibe S., Fuseya T., Tamaya T. (1996). GTP-binding protein and its associated event in membranes from endometrial carcinoma. Oncol. Rep..

[45] Imai A., Horibe S., Takagi A., Tamaya T. (1997). Gi protein activation of gonadotropin-releasing hormone-mediated protein dephosphorylation in human endometrial carcinoma. Am. J. Obs. Gynecol..

[46] Imai A., Takagi A., Horibe S., Takagi H., Tamaya T. (1998). Fas and Fas ligand system may mediate antiproliferative activity of gonadotropin-releasing hormone receptor in endometrial cancer cells. Int. J. Oncol..

[47] Ozturk H.B., Vural B., Caliskan E., Solakoglu S. (2010). Effect of GnRH analogues and octreotide treatment on apoptosis and the cell proliferation of endometrium adenocarcinoma cell lines. J. Turk. Ger. Gynecol. Assoc..

[48] Walters K., Chin Y.P., Wu T.J. (2007). A processed metabolite of luteinizing hormone-releasing hormone has proliferative effects in endometrial cells. Am. J. Obs. Gynecol..

[49] Cho-Clark M., Larco D.O., Semsarzadeh N.N., Vasta F., Mani S.K., Wu T.J. (2014). GnRH-(1-5) transactivates EGFR in Ishikawa human endometrial cells via an orphan G protein-coupled receptor. Mol. Endocrinol..

[50] Engel J.B., Hahne J.C., Hausler S.F., Meyer S., Segerer S.E., Diessner J., Dietl J., Honig A. (2012). Peptidomimetic GnRH antagonist AEZS-115 inhibits the growth of ovarian and endometrial cancer cells. Anticancer Res..

[51] Sealfon S.C., Weinstein H., Millar R.P. (1997). Molecular mechanisms of ligand interaction with the gonadotropin-releasing hormone receptor. Endocr. Rev..

[52] Millar R., Lowe S., Conklin D., Pawson A., Maudsley S., Troskie B., Ott T., Millar M., Lincoln G., Sellar R. (2001). A novel mammalian receptor for the evolutionarily conserved type II GnRH. Proc. Natl. Acad. Sci. USA.

[53] Neill J.D., Duck L.W., Sellers J.C., Musgrove L.C. (2001). A gonadotropin-releasing hormone (GnRH) receptor specific for GnRH II in primates. Biochem. Biophys. Res. Commun..

[54] Gründker C., Günthert A.R., Millar R.P., Emons G. (2002). Expression of gonadotropin-releasing hormone II (GnRH-II) receptor in human endometrial and ovarian cancer cells and effects of GnRH-II on tumor cell proliferation. J. Clin. Endocrinol. Metab..

[55] Gründker C., Schlotawa L., Viereck V., Eicke N., Horst A., Kairies B., Emons G. (2004). Antiproliferative effects of the GnRH antagonist cetrorelix and of GnRH-II on human endometrial and ovarian cancer cells are not mediated through the GnRH type I receptor. Eur. J. Endocrinol..

[56] Eicke N., Günthert A.R., Viereck V., Siebold D., Béhé M., Becker T., Emons G., Gründker C. (2005). GnRH-II receptor-like antigenicity in human placenta and in cancers of the human reproductive organs. Eur. J. Endocrinol..

[57] Eicke N., Gunthert A.R., Emons G., Grundker C. (2006). GnRH-II agonist [D-Lys6]GnRH-II inhibits the EGF-induced mitogenic signal transduction in human endometrial and ovarian cancer cells. Int. J. Oncol..

[58] Fister S., Gunthert A.R., Emons G., Grundker C. (2007). Gonadotropin-releasing hormone type II antagonists induce apoptotic cell death in human endometrial and ovarian cancer cells in vitro and in vivo. Cancer Res..

[59] Fister S., Gunthert A.R., Aicher B., Paulini K.W., Emons G., Grundker C. (2009). GnRH-II antagonists induce apoptosis in human endometrial, ovarian, and breast cancer cells via activation of stress-induced MAPKs p38 and JNK and proapoptotic protein Bax. Cancer Res..

[60] Kim K.Y., Choi K.C., Park S.H., Chou C.S., Auersperg N., Leung P.C. (2004). Type II gonadotropin-releasing hormone stimulates p38 mitogen-activated protein kinase and apoptosis in ovarian cancer cells. J. Clin. Endocrinol. Metab..

[61] Grundker C., Volker P., Griesinger F., Ramaswamy A., Nagy A., Schally A.V., Emons G. (2002). Antitumor effects of the cytotoxic luteinizing hormone-releasing hormone analog AN-152 on human endometrial and ovarian cancers xenografted into nude mice. Am. J. Obs. Gynecol..

[62] Schally A.V., Nagy A. (1999). Cancer chemotherapy based on targeting of cytotoxic peptide conjugates to their receptors on tumors. Eur. J. Endocrinol..

[63] Westphalen S., Kotulla G., Kaiser F., Krauss W., Werning G., Elsasser H.P., Nagy A., Schulz K.D., Grundker C., Schally A.V. (2000). Receptor mediated antiproliferative effects of the cytotoxic LHRH agonist AN-152 in human ovarian and endometrial cancer cell lines. Int. J. Oncol..

[64] Günthert A.R., Gründker C., Bongertz T., Schlott T., Nagy A., Schally A.V., Emons G. (2004). Internalization of cytotoxic analog AN-152 of luteinizing hormone-releasing hormone induces apoptosis in human endometrial and ovarian cancer cell lines independent of multidrug resistance-1 (MDR-1) system. Am. J. Obs. Gynecol..

[65] Nechushtan A., Yarkoni S., Marianovsky I., Lorberboum-Galski H. (1997). Adenocarcinoma cells are targeted by the new GnRH-PE66 chimeric toxin through specific gonadotropin-releasing hormone binding sites. J. Biol Chem..

[66] Palyi I., Vincze B., Lovas S., Mezo I., Pato J., Kalnay A., Turi G., Gaal D., Mihalik R., Peter I. (1999). Gonadotropin-releasing hormone analogue conjugates with strong selective antitumor activity. Proc. Natl. Acad. Sci. USA.

[67] Li X., Shen B., Chen Q., Zhang X., Ye Y., Wang F., Zhang X. (2016). Antitumor effects of cecropin B-LHRH′ on drug-resistant ovarian and endometrial cancer cells. BMC Cancer.

[68] Grundker C., Huschmand Nia A., Emons G. (2005). Gonadotropin-releasing hormone receptor-targeted gene therapy of gynecologic cancers. Mol. Cancer.

[69] Perez-Medina T., Bajo J., Folgueira G., Haya J., Ortega P. (1999). Atypical endometrial hyperplasia treatment with progestogens and gonadotropin-releasing hormone analogues: Long-term follow-up. Gynecol. Oncol..

[70] Minig L., Franchi D., Boveri S., Casadio C., Bocciolone L., Sideri M. (2011). Progestin intrauterine device and GnRH analogue for uterus-sparing treatment of endometrial precancers and well-differentiated early endometrial carcinoma in young women. Ann. Oncol..

[71] Pashov A.I., Tskhay V.B., Ionouchene S.V. (2012). The combined GnRH-agonist and intrauterine levonorgestrel-releasing system treatment of complicated atypical hyperplasia and endometrial cancer: A pilot study. Gynecol. Endocrinol..

[72] Pronin S.M., Novikova O.V., Andreeva J.Y., Novikova E.G. (2015). Fertility-sparing treatment of early endometrial cancer and complex atypical hyperplasia in young women of childbearing potential. Int J. Gynecol. Cancer.

[73] Zhou H., Cao D., Yang J., Shen K., Lang J. (2017). Gonadotropin-releasing hormone agonist combined with a levonorgestrel-releasing intrauterine system or letrozole for fertility-preserving treatment of endometrial carcinoma and complex atypical hyperplasia in young women. Int. J. Gynecol. Cancer.

[74] Zhang Z., Huang H., Feng F., Wang J., Cheng N. (2019). A pilot study of gonadotropin-releasing hormone agonist combined with aromatase inhibitor as fertility-sparing treatment in obese patients with endometrial cancer. J. Gynecol. Oncol..

[75] Tock S., Jadoul P., Squifflet J.L., Marbaix E., Baurain J.F., Luyckx M. (2018). fertility sparing treatment in patients with early stage endometrial cancer, using a combination of surgery and GnRH agonist: A monocentric retrospective study and review of the literature. Front. Med..

[76] Yin J., Ma S., Shan Y., Wang Y., Li Y., Jin Y., Pan L. (2020). Risk factors for recurrence in patients with atypical endometrial hyperplasia and endometrioid adenocarcinoma after fertility-sparing treatments. Cancer Prev. Res..

[77] Gallagher C.J., Oliver R.T., Oram D.H., Fowler C.G., Blake P.R., Mantell B.S., Slevin M.L., Hope-Stone H.F. (1991). A new treatment for endometrial cancer with gonadotrophin releasing-hormone analogue. Br. J. Obs. Gynaecol..

[78] Jeyarajah A.R., Gallagher C.J., Blake P.R., Oram D.H., Dowsett M., Fisher C., Oliver R.T. (1996). Long-term follow-up of gonadotrophin-releasing hormone analog treatment for recurrent endometrial cancer. Gynecol. Oncol..

[79] Covens A., Thomas G., Shaw P., Ackerman I., Osborne R., Lukka H., Carey M., Franssen E., Roche K. (1997). A phase II study of leuprolide in advanced/recurrent endometrial cancer. Gynecol. Oncol..

[80] Lhomme C., Vennin P., Callet N., Lesimple T., Achard J.L., Chauvergne J., Luporsi E., Chinet-Charrot P., Coudert B., Couette J.E. (1999). A multicenter phase II study with triptorelin (sustained-release LHRH agonist) in advanced or recurrent endometrial carcinoma: A French anticancer federation study. Gynecol. Oncol..

[81] Noci I., Borri P., Bonfirraro G., Chieffi O., Arcangeli A., Cherubini A., Dabizzi S., Buccoliero A.M., Paglierani M., Taddei G.L. (2001). Longstanding survival without cancer progression in a patient affected by endometrial carcinoma treated primarily with leuprolide. Br. J. Cancer.

[82] Asbury R.F., Brunetto V.L., Lee R.B., Reid G., Rocereto T.F., Gynecologic Oncology G. (2002). Goserelin acetate as treatment for recurrent endometrial carcinoma: A gynecologic oncology group study. Am. J. Clin. Oncol..

[83] Emons G., Kaufmann M., Gorchev G., Tsekova V., Grundker C., Gunthert A.R., Hanker L.C., Velikova M., Sindermann H., Engel J. (2010). Dose escalation and pharmacokinetic study of AEZS-108 (AN-152), an LHRH agonist linked to doxorubicin, in women with LHRH receptor-positive tumors. Gynecol. Oncol..

[84] Miller D.S., Scambia G., Bondarenko I., Westermann A.M., Oaknin A., Oza A.M., Lisyanskaya A.S., Vergote I., Wenham R.M., Temkin S.M. (2018). ZoptEC: Phase III randomized controlled study comparing zoptarelin with doxorubicin as second line therapy for locally advanced, recurrent, or metastatic endometrial cancer (NCT01767155). J. Clin. Oncol..

[85] ClinicalTrials.gov Zoptarelin Doxorubicin (AEZS 108) as Second Line Therapy for Endometrial Cancer (ZoptEC). https://clinicaltrials.gov/ct2/show/results/NCT01767155.

[86] Grundker C., Emons G. (2017). The role of gonadotropin-releasing hormone in cancer cell proliferation and metastasis. Front. Endocrinol..

